# The Utility of Dot Phrases and SmartPhrases in Improving Physician Documentation of Interpreter Use

**DOI:** 10.5811/westjem.18352

**Published:** 2024-04-02

**Authors:** Katrin Jaradeh, Elaine Hsiang, Malini K. Singh, Christopher R. Peabody, Steven Straube

**Affiliations:** * Department of Emergency Medicine, University of California, San Francisco, California; † San Francisco General Hospital, San Francisco, California

## Abstract

**Background:**

Patients with limited English proficiency (LEP) experience significant healthcare disparities. Clinicians are responsible for using and documenting their use of certified interpreters for patient encounters when appropriate. However, the data on interpreter use documentation in the emergency department (ED) is limited and variable. We sought to assess the effects of dot phrase and SmartPhrase implementation in an adult ED on the rates of documentation of interpreter use.

**Methods:**

We conducted an anonymous survey asking emergency clinicians to self-report documentation of interpreter use. We also retrospectively reviewed documentation of interpreter- services use in ED charts at three time points: 1) pre-intervention baseline; 2) post-implementation of a clinician-driven dot phrase shortcut; and 3) post-implementation of a SmartPhrase.

**Results:**

Most emergency clinicians reported using an interpreter “almost always” or “often.” Our manual audit revealed that at baseline, interpreter use was documented in 35% of the initial clinician note, 4% of reassessments, and 0% of procedure notes; 52% of discharge instructions were written in the patients’ preferred languages. After implementation of the dot phrase and SmartPhrase, respectively, rates of interpreter-use documentation improved to 43% and 97% of initial clinician notes, 9% and 6% of reassessments, and 5% and 35% of procedure notes, with 62% and 64% of discharge instructions written in the patients’ preferred languages.

**Conclusion:**

There was a discrepancy between reported rates of interpreter use and interpreter-use documentation rates. The latter increased with the implementation of a clinician-driven dot phrase and then a SmartPhrase built into the notes. Ensuring accurate documentation of interpreter use is an impactful step in language equity for LEP patients.

Population Health Research Capsule What do we already know about this issue?

*Patients with limited English proficiency experience healthcare disparities. Using interpreters reduces unnecessary testing and hospitalizations for this population.*

What was the research question?

*Does implementing a dot phrase and SmartPhrase increase documentation of interpreter use?*

What was the major finding of the study?

*Documentation of interpreter use in the history and physical rose from 35% to 43% (dot phrase) and then to 97% (SmartPhrase).*

How does this improve population health?

*An intervention to improve documentation of interpreter use helps ensure language equity for limited English proficiency patients.*

## INTRODUCTION

As of 2019, over 65 million people in the United States (US) speak a language other than English, with approximately 20% of households reporting speaking English less than “very well,” also known as limited-English proficiency (LEP).^1^ In the US, presidential Executive Order 13166, enacted in 2000, ensures that LEP patients are offered interpretation services at healthcare facilities receiving federal assistance.^2^^,^^3^ The lack of access to language-concordant care contributes to healthcare disparities among LEP patients.^4^

In the emergency department (ED), LEP patients were more likely to have unplanned revisits within 72 hours^5^ with limited evidence suggesting differences in triage or admission decisions depending on interpreter use.^6^ Recent data demonstrates increased unnecessary testing and hospital admission with longer lengths of stay among LEP patients who did not receive professional interpreting services.^7^^,^^8^ Documentation of interpreter use is often used as a proxy for interpreter use. Several groups of researchers studied the rate of interpreter-use documentation in the hospital. One found that 41% (30/74) of patients had a consent form in their native language or that an interpreter had signed it.^8^

Interventions have been implemented to improve documentation of interpreter use. Bender et al found that when they placed flyers in the ED and made pre-work shift announcements, documentation of interpreter use increased from a baseline rate of 5% to 25%.^9^ In 2021, a study among patients admitted to a pediatric service found that using a dot phrase increased interpreter use from 64% to 81%, and interpreter-use documentation increased from 69% to 98%.^10^ To our knowledge, there have been no studies investigating the use of a dot phrase (text inserted with keyboard shortcuts) or a SmartPhrase (abbreviations or words used to pull long phrases into a physician’s note) in an adult ED to improve documentation of interpreter use. We assessed the effects of a dot phrase and a SmartPhrase in an adult ED on the rates of documentation of interpreter use. We hypothesized these interventions would increase documentation rates.

## METHODS

We conducted this study at a Level I academic trauma center in an adult ED, where interpreters are available over the phone 24/7 and in person during designated hours. First, we gathered patients’ medical record numbers (MRN) from interpreter services that documented an interpreter had been used. A pre-intervention retrospective chart review was conducted to assess the baseline rate of interpreter-use documentation in the electronic health record (EHR). Second, we surveyed emergency clinicians to assess their perspective on interpreter use and documentation. Third, we implemented a dot phrase and then a SmartPhrase and retrospectively reviewed charts for documentation of interpreter use. Both instruments were being developed at the same time, but the dot phrase was completed more quickly and implemented first. Documentation of interpreter use was captured within the history and physical (H&P), reassessments, procedure notes, and discharge instructions (DCI), which includes a verbal discussion, written instructions, and attachments. We excluded charts from the study if the patient only spoke or preferred to speak in English, left without being seen, MRNs were not found, or if it it was a duplicate record. There was no prior training on documentation of interpreter use. We analyzed data using descriptive statistics. This study was deemed exempt by our institutional review board.

### Pre-Intervention

We verified MRNs from the interpreter service data in the EHR. A number generator was used to randomize and identify patients for chart review. To minimize clinician-specific practice patterns, we audited one chart per day from July–September 2021 from various shift times to estimate the pre-intervention rate of interpreter use documentation.

### Clinician Survey

We emailed an anonymous survey to 128 ED attendings, fellows, residents, and nurse practitioners regarding interpreter-use documentation after the pre-intervention data was collected. One follow-up email was sent. We created a survey of 14 multiple-choice questions hosted on Qualtrics (Qualtrics, Provo, UT). The survey included demographics, questions about interpreter use, documentation, and ways to improve documentation.

### Dot Phrase

A dot phrase is a block of text inserted using a keyboard shortcut proceeded by a dot that facilitates clinician’s documentation. Clinicians can input the phrase “.EDinterpreter” for the statement “A [phone, in-person] [language options] interpreter was used on [date and time], [INTERPRETER ID #]” to be added in the EHR. The dot phrase was available on July 1, 2022. All charts from interpreter services data were audited between July 1–October 14, 2022.

### SmartPhrase

We embedded a SmartPhrase into the H&P and procedure notes creating a “hard stop” where clinicians could not sign their notes until the SmartPhrase was completed. This could be bypassed by deleting the SmartPhrase. If a non-English language was selected, the SmartPhrase would prompt to choose the patient’s preferred language. The SmartPhrase was available on November 1, 2022. All charts from interpreter services data were audited between November 1–February 1, 2023.

## RESULTS

### Pre-Intervention

**Figure 1.**
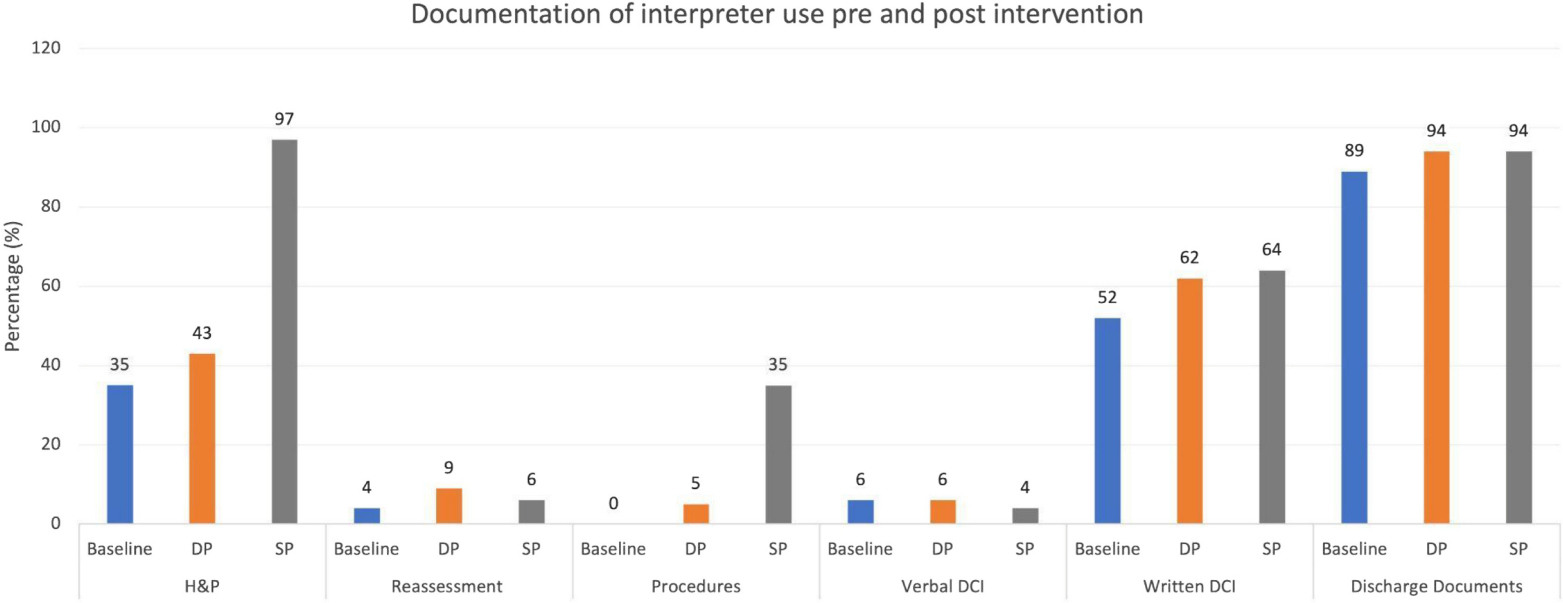
Percentage of patient charts with documentation of interpreter use at baseline (blue), after the creation of the dot phrase (orange), and after the creation of the SmartPhrase (gray). *H&P*, history and physical; *DCI*, discharge instructions; *DP*, dot phrase; *SP*, SmartPhrase and procedure note implementation.

Of 91 audited charts, Spanish (61%) was the most preferred language, followed by Cantonese/ Mandarin/ Taishanese (37%), and Russian (2%). Use of an interpreter was documented in 35% of H&Ps, 4% of reassessments, and in 0% of procedure notes. Within the discharge instructions, 6% of charts indicated discussing instructions using an interpreter; 52% of written DCIs and 89% of attachments were provided in the patient’s native language (Figure 1).

### Clinician Survey

**Figure 2.**
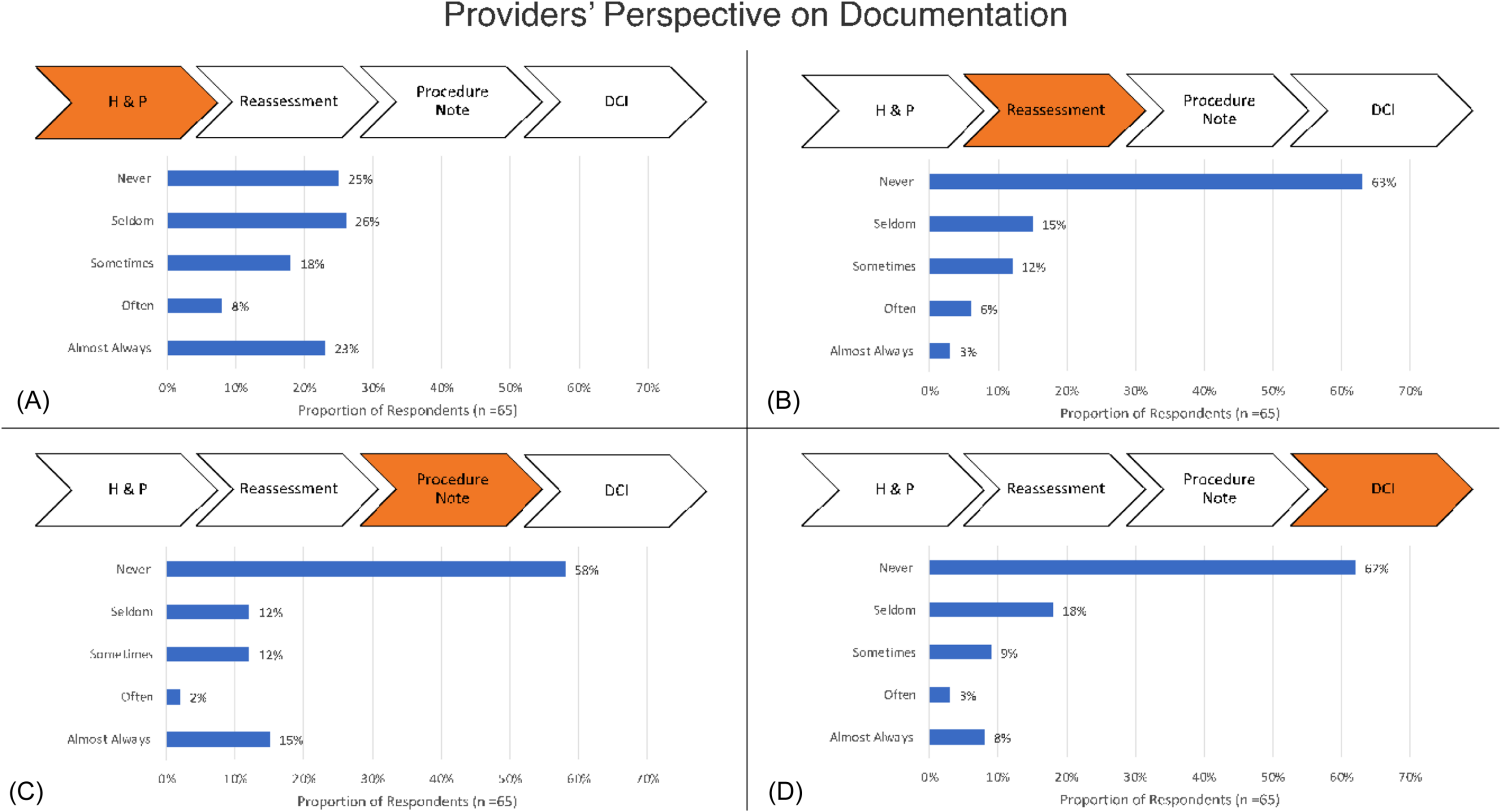
(a) Emergency clinicians’ perspective on documentation of interpreter use in the history and physical. (b) Emergency clinicians’ perspective on documentation of interpreter use in the reassessment. (c) Emergency clinicians’ perspective on documentation of interpreter use in the procedure note. (d) Emergency clinicians’ perspective on documentation of interpreter use in the DCI. *H&P*, history and physical; *DCI*, discharge instructions.

Of 128 emergency clinicians who received the survey, 67 (52%) initiated and 65 (51%) completed it. Of the respondents, 46% were residents, 37% attendings, 9% NPs, and 8% fellows. Clinicians reported use of an interpreter “almost always” or “often” 66% and 25% of the time when interacting with LEP patients. Additionally, 23% and 8% of clinicians reported “almost always” or “often” documenting use of an interpreter in the H&P (Figure 2a). Clinicians reported “almost always” documenting use of an interpreter in the reassessment (3%), procedure (15%), and DCI (8%) portions of the note (Figure 2b, 2c, 2d). When asked what can make documentation easier, 41% suggested additions to the ED note template with 29% recommending a dot phrase.

### Dot Phrase

Of 866 audited charts, we analyzed 809 (93%). Spanish (67%) was the most preferred language, followed by Cantonese/Mandarin/Taishanese (32%), and Russian (1%). Forty-three percent of H&Ps, 9% of reassessments, and 5% of procedure notes had documentation of interpreter use. Documentation of interpreter use during discharge remained at 6%. The written portion and attachments of the DCI were in the patient’s native language in 62% and 94% of charts.

### SmartPhrase

Of 779 audited charts, we analyzed 646 (83%). Spanish (64%) was the most preferred language, followed by Cantonese/Mandarin/Taishanese (35%), and Russian (0.62%). Ninety-seven percent of H&P, 6% of the reassessments, and 35% of procedure notes had documentation of interpreter use. Regarding the verbal DCI, 4% documented interpreter use. The written portion and attachments were in the patient’s native language in 64% and 94% of charts (Figure 1).

## DISCUSSION

Documentation rates of interpreter use increased after implementation of a dot phrase and a SmartPhrase. After implementing the SmartPhrase, almost 100% of the H&Ps and 35% of procedure notes documented interpreter use. Because the SmartPhrase was embedded only in H&Ps and procedure notes, we did not expect increases in the DCI and reassessments. The general rates of interpreter-use documentation in this study and previous studies vary. Behairy et al found that at their children’s hospital documentation of interpreter use was 0%,^11^ whereas Taira et al found documentation of interpreter use in their public ED to be 4.6%.^12^ To our knowledge, this is one of the first studies on the impact of a dot phrase and a SmartPhrase on documentation of interpreter use in an adult ED.

There was a discrepancy between reported rates of interpreter use and documentation of interpreter use. Despite 66% of clinicians reporting “almost always” using an interpreter, only 23% reported “almost always” documenting their use in the H&P. The same discrepancy was seen among reassessments (3%), procedure notes (35%), and DCIs (8%) where clinicians reported they “almost always” documented their use. While we did not specifically ask clinicians when they use an interpreter (while gathering the H&P, etc, setting documentation as a proxy for interpreter use, many clinicians speaking to their patients with an interpreter would not have the documentation to support their claim. Lastly, clinicians may have used an ad hoc interpreter (family member or a member of the healthcare team), as the survey did not specify use of professional interpretation. This may account for some of the discrepancy between the reported and actual rates of interpreter use per interpreter services data.

Next, we hope to assess the impact of improved documentation on patient care.

## LIMITATIONS

This was a single-institution study and results may not be generalizable. Variability in documentation among emergency clinicians, and in time and day of shift were not captured. Since only one chart per day was reviewed for pre-intervention data, documentation rates may have been more influenced by time of day than post-intervention rates, affecting the differences in pre-/post-intervention changes. We did not track the data of dot phrase and SmartPhrase use. Further, despite the SmartPhrase leading to a “hard stop,” clinicians could delete the SmartPhrase. However, we included both the dot phrase and SmartPhrase as interventions since clinicians could add the dot phrase into other elements of the EHR when they used an interpreter (eg, reassessments).

## CONCLUSION

Documentation of interpreter use is varied. There was a discrepancy between reported rates of interpreter use and interpreter-use documentation. Implementation of a dot phrase and a SmartPhrase improved documentation of interpreter use, suggesting its feasibility to improve clinician documentation.
